# Systemic Administration of Antiretrovirals Prior to Exposure Prevents Rectal and Intravenous HIV-1 Transmission in Humanized BLT Mice

**DOI:** 10.1371/journal.pone.0008829

**Published:** 2010-01-21

**Authors:** Paul W. Denton, John F. Krisko, Daniel A. Powell, Melissa Mathias, Youn Tae Kwak, Francisco Martinez-Torres, Wei Zou, Deborah A. Payne, Jacob D. Estes, J. Victor Garcia

**Affiliations:** 1 Department of Internal Medicine, University of Texas Southwestern Medical Center at Dallas, Dallas, Texas, United States of America; 2 Department of Pathology, University of Texas Southwestern Medical Center at Dallas, Dallas, Texas, United States of America; 3 The AIDS and Cancer Virus Program, SAIC-Frederick, Inc, National Cancer Institute, Frederick, Maryland, United States of America; McGill University AIDS Centre, Canada

## Abstract

Successful antiretroviral pre-exposure prophylaxis (PrEP) for mucosal and intravenous HIV-1 transmission could reduce new infections among targeted high-risk populations including discordant couples, injection drug users, high-risk women and men who have sex with men. Targeted antiretroviral PrEP could be particularly effective at slowing the spread of HIV-1 if a single antiretroviral combination were found to be broadly protective across multiple routes of transmission. Therefore, we designed our *in vivo* preclinical study to systematically investigate whether rectal and intravenous HIV-1 transmission can be blocked by antiretrovirals administered systemically prior to HIV-1 exposure. We performed these studies using a highly relevant *in vivo* model of mucosal HIV-1 transmission, humanized Bone marrow/Liver/Thymus mice (BLT). BLT mice are susceptible to HIV-1 infection via three major physiological routes of viral transmission: vaginal, rectal and intravenous. Our results show that BLT mice given systemic antiretroviral PrEP are efficiently protected from HIV-1 infection regardless of the route of exposure. Specifically, systemic antiretroviral PrEP with emtricitabine and tenofovir disoproxil fumarate prevented both rectal (Chi square = 8.6, df = 1, p = 0.003) and intravenous (Chi square = 13, df = 1, p = 0.0003) HIV-1 transmission. Our results indicate that antiretroviral PrEP has the potential to be broadly effective at preventing new rectal or intravenous HIV transmissions in targeted high risk individuals. These *in vivo* preclinical findings provide strong experimental evidence supporting the potential clinical implementation of antiretroviral based pre-exposure prophylactic measures to prevent the spread of HIV/AIDS.

## Introduction

Preventing the spread of HIV to new individuals is critical to stopping the HIV/AIDS pandemic. However, few successful strategies to prevent HIV transmissions currently exist [1]–[5]. Novel approaches to prevent HIV transmission, including effective vaccines, are being considered and developed [6]. In particular, antiretroviral pre-exposure prophylaxis (PrEP) has been postulated to be a potentially highly effective prevention modality [7]–[15]. There are many reasons to consider implementing targeted antiretroviral PrEP until less toxic, easier to deliver and more potent prevention methods become available. Candidate antiretroviral drugs for PrEP already exist. Antiretrovirals to prevent vertical HIV transmission are already used clinically. In 2007, 500,000 (∼33%) HIV positive pregnant women worldwide received antiretrovirals to prevent HIV transmission between them and their children [5]. Additionally, PrEP is a prevention approach that can be discretely utilized without requiring partner consent. While PrEP comes with associated costs, these should not distract from the vast prospective positive impact of PrEP: targeted PrEP has been mathematically modeled to avert up to 3 million new infections over a 10 year period in Sub-Saharan Africa alone [16]. Antiretroviral PrEP could benefit numerous groups at risk of either vaginal, rectal or intravenous HIV exposure: discordant couples, high risk women, men who have sex with men and injection drug users [13]. Topical microbicides may be identified that block mucosal HIV transmission [17]–[19]. However, as illustrated by several setbacks in recent clinical trials (i.e. Microbicides Development Program study 301using 0.5% Pro 2000/5) microbicide development and implementation lags far behind that of clinically approved antiretrovirals [13], [20], [21]. In addition, it should be noted that topical interventions will not prevent intravenous HIV transmission. The diversity of the groups targeted for PrEP highlights the need for broad prevention modalities that protect from the multiple and frequently overlapping ways by which an individual may become exposed to HIV.

We performed comprehensive efficacy studies to determine whether a single antiretroviral PrEP approach can protect from multiple routes of HIV transmission using a uniform and highly relevant experimental platform. When choosing a model system to perform PrEP efficacy studies, it was important to identify critical characteristics that the system would have to exhibit in order to study HIV prevention modalities. Such a model would permit studying the interplay between *de novo* generated human immune cells and HIV being transmitted via physiological routes in the context of highly active antiretroviral drugs. In addition, such a model should be affordable, available to many investigators and capable of providing relatively rapid feedback on the efficacy of any intervention being evaluated. To this end, we chose the humanized Bone marrow/Liver/Thymus (BLT) mouse as our experimental system [22].

Humanized BLT mice are individually bioengineered to exhibit a complete, systemic, self-renewing reconstitution of all major human hematopoietic lineages including T, B, monocyte/macrophage, dendritic and natural killer cells that facilitates the generation of functional human immune responses [22]–[26]. The levels of HIV receptor and co-receptor expression in BLT mice reflect those observed in humans and the pathogenesis of CCR5 tropic HIV-1 in BLT mice mirrors descriptions of HIV pathogenesis in infected individuals [24], [26]. Particularly relevant to this study is the broad and systemic reconstitution of BLT mice with human immune cells necessary for HIV-1 replication and transmission (CD4^+^ T cells, macrophages and dendritic cells) that encompasses the peripheral blood and the rectal and vaginal mucosa rendering BLT mice susceptible to intravenous and mucosal HIV-1 infection [22], [24], [26]. Furthermore, systemic PrEP with a combination of antiretrovirals (FTC: emtricitabine and TDF: tenofovir disoproxil fumarate) prevents vaginal HIV-1 transmission in BLT mice establishing this model as a novel system for *in vivo* preclinical evaluation of HIV prevention modalities [24].

To date, the majority of HIV prevention research has focused on the assessment of the safety and effectiveness of products capable of preventing HIV transmission via the vaginal compartment. Receptive anal intercourse is common among men who have sex with men and rectal transmission is a major driving force of the AIDS pandemic [5]. In addition, rectal transmission is also likely to account for a significant number of transmissions to women [27]. We hypothesized that systemic antiretroviral PrEP can provide protection from rectal and intravenous HIV-1 transmission. We tested this hypothesis by treating BLT mice systemically with FTC/TDF prior to exposure and we determined that antiretroviral PrEP can prevent rectal and intravenous HIV-1 transmission. Our *in vivo* preclinical efficacy data shows that systemic antiretroviral PrEP provides strong protection against HIV-1 infection regardless of the route of transmission.

## Materials and Methods

### Preparation of Humanized BLT Mice, Tissue Harvesting and Microscopic and Flow Cytometric Analyses

BLT mice were prepared essentially as previously described [22]–[26]. Briefly, thy/liv implanted [28] NOD/SCID or NOD/SCID-gamma chain null mice (The Jackson Laboratories, Bar Harbor, ME) were transplanted with autologous human fetal liver CD34^+^ cells (Advanced Bioscience Resources, Alameda, CA) and monitored for human reconstitution in peripheral blood by flow cytometry [22], [24], [26]. Mice were maintained at the Animal Resources Center of University of Texas Southwestern Medical Center (UTSWMC) in accordance with protocols approved by the UTSWMC Institutional Animal Care and Use Committee. Tissues were harvested and then evaluated by molecular, microscopic and flow cytometric analyses for evidence of HIV infection as we have previously described [22], [24], [26]. Briefly, minced and/or digested tissues were disrupted and filtered through a 70 µm cell strainer. Liver and lung mononuclear cells were isolated using a Percoll gradient. In other tissues, red blood cells were lysed (ACK lysing buffer). Once isolated, mononuclear cells were washed, enumerated and utilized in the indicated assays described below.

### Systemic Application of FTC/TDF and Exposure of BLT Mice to HIV-1

**Figure 1:**
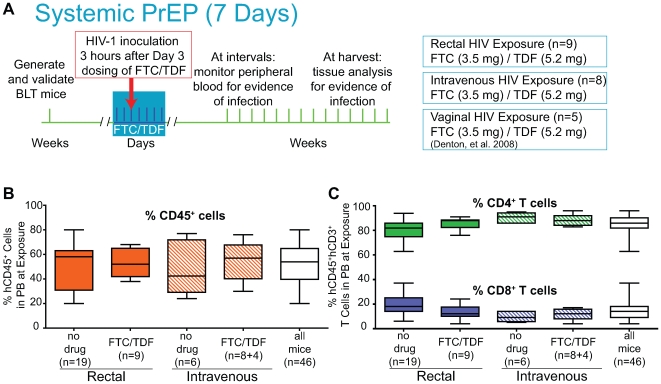
Experimental design and reconstitution of BLT mice with human hematopoietic cells. (A) Systemic PrEP with FTC/TDF (daily administrations for 7 consecutive days) to prevent rectal, intravenous and vaginal HIV-1 transmission. Viral exposure was performed 3 hours following the third FTC/TDF dosing. (B) Peripheral blood human leukocytes (CD45^+^) levels in each of the groups of BLT mice used. (C) Peripheral blood human T lymphocytes (CD4^+^ and CD8^+^) levels in each of the groups of BLT mice used. Box-plot interpretation for this and subsequent figures: middle line is the median; box extends from the 25^th^ to the 75^th^ percentiles; error bars extend down to the lowest value and up to the highest value.

Stocks of HIV-1_JR-CSF_ [29] were prepared, titered and p24 content was determined as we have previously described [30], [31]. Briefly, virus supernatants were collected following transient transfection of 293T cells with the plasmid molecular clone of JR-CSF. Supernatant p24 content was determined by ELISA (Coulter, kit sensitivity: 7.8 pg/ml). HIV-1 exposures were performed essentially as previously described using a total volume of 2–10 µL (rectal: 170 ng p24) or 200 µL (intravenous: 58 ng p24) [26], [32]. Intravenous HIV-1 exposures were administered via the tail vein. FTC/TDF dosing was based on published efficacy in BLT mice [24]. To prepare FTC/TDF for BLT mouse administration Truvada® capsules (Gilead, Foster City, CA) were dissolved in deionized water with 10% DMSO then sterile filtered (0.22µm). FTC and TDF concentrations were initially estimated by UV spectrophotometry and then confirmed by mass spectroscopy (UTSW Chemistry Core). The FTC/TDF solution was administered intraperitoneally (daily injections of 3.5 mg FTC and 5.2 mg TDF) prior or subsequent to exposure to HIV-1, as indicated in Figure 1A and the text [24], [33]–[35].

### Analysis of HIV-1 Infection of BLT Mice

In this study, the primary endpoint was determining whether a given intervention protected BLT mice from HIV-1 transmission. To ensure that the most stringent criteria were met by the intervention, we designed a high threshold defining “protection”. We defined “protection” in treated groups as the complete absence of any evidence of infection, such that protected mice had no positive results for the presence of HIV by any method of analysis at any time point tested. A positive result for the presence of HIV-1 from any treated animal by any method indicated a lack of protection referred to as “breakthrough” infection.

Infection of BLT mice with HIV-1 was monitored in peripheral blood by determining plasma levels of viral antigenemia (ELISA p24, Coulter, assay sensitivity: 7.8 pg/ml), levels of viral RNA in plasma (Amplicor, Roche, assay sensitivity of 400 RNA copies per ml) and levels of viral DNA in peripheral blood cells (real time PCR analysis, assay sensitivity of 10 copies) as previously described [22], [24], [26], [31]. Analysis for systemic infection was performed on tissues harvested from infected mice or on cells isolated from the indicated tissues utilizing in situ hybridization, real time PCR analysis and co-culture with PHA activated allogeneic human PBMC as previously described [22], [24], [26]. In the case of breakthrough infection, it is possible that any developed drug resistance mutants could revert back to wild-type in the absence of drug selection following the completion of PrEP. Therefore, to increase our likelihood of detecting any developed resistance mutants we performed our sequence analysis on DNA samples from the earliest possible time point at which HIV-1 DNA was detected. We directly sequenced the entire reverse transcriptase gene from cell-associated HIV-1 DNA amplification products to evaluate whether these transmission events resulted from drug resistant variants. No described resistance mutations in reverse transcriptase were observed [36]–[39].

### Statistical Analysis

All statistical analyses (alpha level: 0.05) were performed in Prism version 5 (Graph Pad Software, Inc., San Diego, CA). Kaplan-Meyer plots indicate the percentage of animals that are HIV-1 positive in the peripheral blood by each time point. Tick marks on the curves represent the time point at which HIV-1 negative animals were censored from the analysis.

## Results

This study models application of antiretroviral PrEP in a manner that closely resembles planned or ongoing PrEP clinical trials evaluating the efficacy of Truvada® [FTC co-formulated with TDF] [13]. Systemic PrEP models routine daily systemic administration of antiretrovirals (not temporally associated with a specific high-risk event) that continues until a general behavior pattern of high-risk actions ceases. Systemic PrEP dosing in the BLT mice continued for 4 days following exposure to simulate how systemic PrEP is expected to continue beyond the last high risk action for a given period before a person would stop the regimen (Figure 1A). Once BLT mice were generated, but prior to HIV-1 exposure, we analyzed their peripheral blood to determine their reconstitution with human cells and their suitability for these studies. All the humanized BLT mice used for these experiments had high levels of human lymphoid (CD45^+^) cells in their peripheral blood (51.0%±16.8 SD, n = 46) (Figure 1B). In addition, all mice were reconstituted with high levels of human CD4^+^ T cells in peripheral blood (Figure 1C).

### Systemically Administered Antiretroviral PrEP Prevents Rectal HIV-1 Transmission

**Figure 2:**
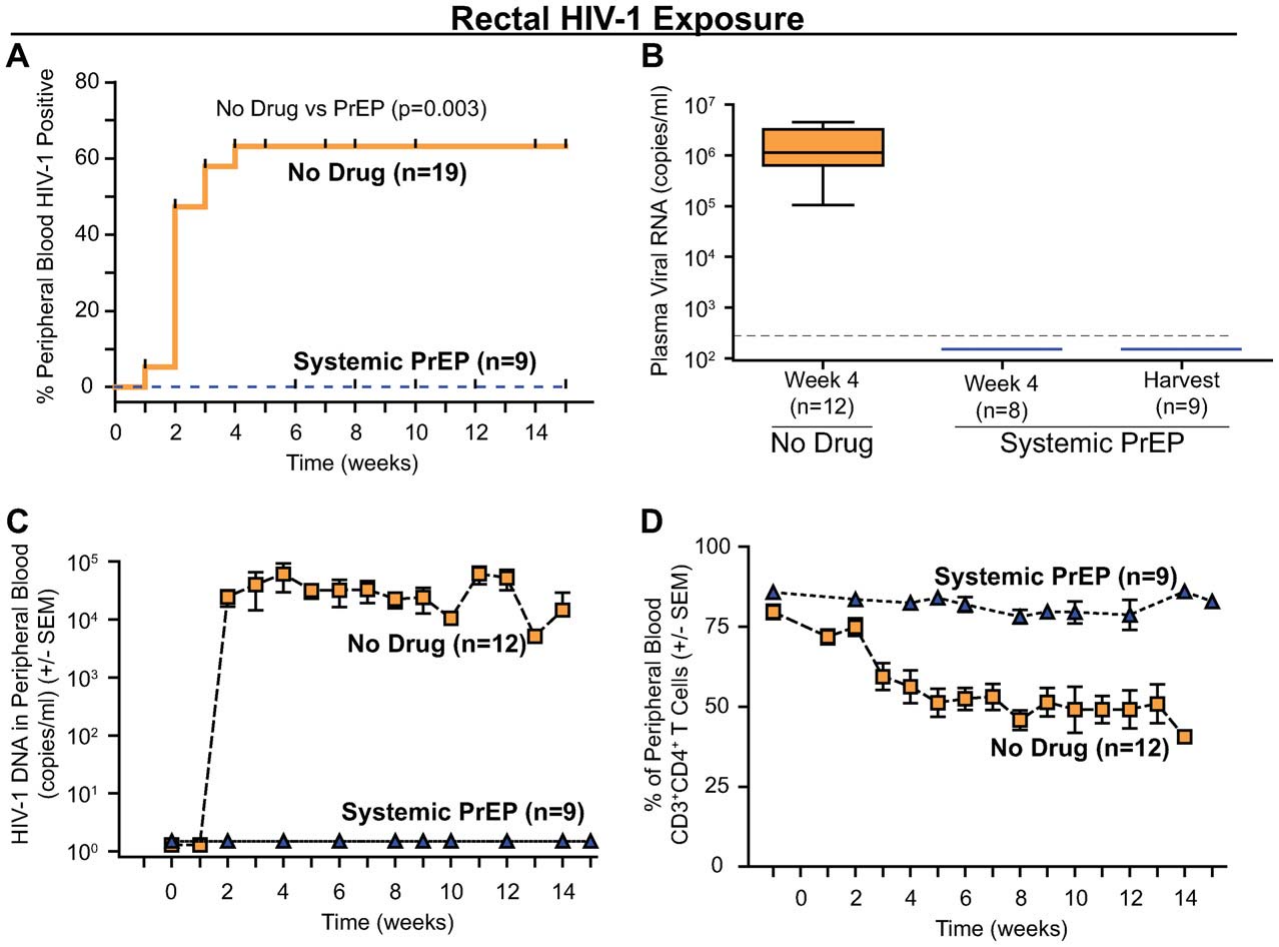
Systemic PrEP with FTC/TDF prevents rectal HIV-1 transmission. (A) Kaplan-Meier plot of the time course to peripheral blood conversion following rectal HIV-1 exposure in BLT mice with or without pre-exposure treatment with systemic FTC/TDF. (B) Plasma viral RNA was only detected in infected non-treated control mice. Mice receiving systemic PrEP were negative for plasma viral RNA. Thin dashed line represents the limit of detection for this assay. (C) PBMC-associated viral DNA was only detected in infected non-treated control mice. Mice receiving systemic PrEP were negative for PBMC-associated viral DNA. (D) Average levels of human CD4^+^ T cells in peripheral blood showed a loss of CD4^+^ T cells in infected non-treated control mice, but not in systemic PrEP treated BLT mice. Note that regardless of the assay utilized there was no evidence of rectal infection in any of the mice receiving systemic PrEP with FTC/TDF.

We have previously shown that systemic PrEP efficiently blocks vaginal HIV-1 transmission in BLT mice [24]. Here we sought to determine whether systemic PrEP also can prevent rectal HIV-1 transmission. As depicted in Figure 1A, test BLT mice (n = 9) were exposed to a single dose of HIV-1 (CCR5-tropic primary isolate JR-CSF [29]) on the third of seven days of consecutive dosing with FTC/TDF. After rectal HIV-1 exposure, BLT mice were followed over time to determine if transmission had occurred. Transmission was defined using most stringent criteria: any single evidence of infection by any method of detection at any of the time points analyzed. Protection was also defined by very stringent criteria: complete lack of evidence of infection, by any method of detection at any of the time points analyzed, including a systemic post-mortem analysis. Using this criteria, none of the samples evaluated from rectally challenged animals also receiving systemic FTC/TDF showed evidence of plasma viral RNA (Amplicor), PBMC-associated HIV-1 DNA (real time PCR), plasma antigenemia (ELISA) or loss of peripheral CD4^+^ T cells (flow cytometry). In contrast, 12 of 19 non-treated control mice became HIV-1 positive (Log-rank [Mantel Cox] Test: Chi square = 8.6, df = 1, p = 0.003) (Figure 2A–D; Table 1). These data indicate that systemically administered antiretrovirals can efficiently prevent rectal HIV-1 transmission in humanized BLT mice.

**Table 1:** Description of BLT mice used to evaluate systemic PrEP for rectal HIV-1 transmission.*

Mouse #		In PB at exposure:		Weeks followed (exposure to harvest)	Peripheral Blood Analysis				Multiple Tissue Analysis		
		% human CD45^+^	% hCD45^+^ hCD3^+^ hCD4^+^		Plasma antigenemia (Gag^p24^)^A^	Plasma viral load (RNA)^B^	PBMC associated viral DNA^C^	Included in Figure 2B–D	In situ hybridization for viral RNA	Quantitative Real time PCR for cell associated viral DNA^C^	Virus rescue of replication competent virus (Gag^p24^)^A^
**No Drug**	1	40	75	3	**Pos (2 of 3)**	**Pos (1 of 1)**	**Pos (1 of 1)**	Yes	**frt,L.int,lu,mln,o,S.int,s,st**	**b,li,lu,o,s**	**b,li,lu,o**
	2	22	76	3	**Pos (2 of 3)**	**Pos (1 of 1)**	**Pos (2 of 2)**	Yes	nd	nd	nd
	3	62	82	3	**Pos (1 of 2)**	**Pos (1 of 1)**	**Pos (1 of 2)**	Yes	**s**	**b,li,lu,o,s**	**b,li,lu,o,s**
	4	43	88	4	**Pos(1 of 2)**	**Pos (1 of 1)**	**Pos (1 of 2)**	Yes	nd	nd	**b,li,lu,s**
	5	69	82	6	**Pos (3 of 3)**	**Pos (1 of 1)**	**Pos (3 of 3)**	Yes	**lu,o,pln,r,s**	**b,li,lu,o,s**	**b,li,lu,o,s**
	6	63	84	7	**Pos (6 of 7)**	**Pos (1 of 1)**	**Pos (5 of 6)**	Yes	**mrt,lu**	**b,li,lu,o,s**	**b,li,lu,o,s**
	7	73	82	7	Neg (0 of 4)	nd	nd	No	nd	nd	nd
	8	27	78	8	Neg (0 of 8)	nd	nd	No	nd	nd	nd
	9	20	82	9	**Pos (5 of 5)**	**Pos (1 of 1)**	**Pos (4 of 5)**	Yes	**s**	**b,li,lu,o,s**	**b,lu,o,s**
	10	64	72	10	Neg (0 of 10)	nd	nd	No	nd	nd	nd
	11	22	71	11	**Pos (11 of 11)**	**Pos (1 of 1)**	**Pos (9 of 10)**	Yes	nd	**b,li,lu,o,s**	nd
	12	63	86	11	Neg (0 of 6)	nd	nd	No	nd	nd	nd
	13	80	86	11	**Pos(7 of 7)**	**Pos (1 of 1)**	**Pos (7 of 7)**	Yes	**s**	**b,li,lu,o,s**	**b,li,lu,o,s**
	14	58	91	12	**Pos (5 of 8)**	**Pos (1 of 1)**	**Pos (8 of 8)**	Yes	**s**	**b,li,lu,o,s**	**b,li,lu,o,s**
	15	59	90	12	Neg (0 of 6)	nd	nd	No	nd	nd	nd
	16	43	76	14	**Pos (13 of 14)**	**Pos (1 of 1)**	**Pos (11 of 13)**	Yes	nd	**b,li,lu,o**	**b,li,lu,o**
	17	39	63	14	**Pos (12 of 14)**	**Pos (1 of 1)**	**Pos (10 of 12)**	Yes	nd	nd	b,li,lu,o,s
	18	31	66	14	Neg (0 of 14)	nd	nd	No	nd	nd	nd
	19	61	94	15	Neg (0 of 9)	nd	nd	No	s	nd	nd
**FTC/TDF**	20	60	87	4	Neg (0 of 2)	Neg (0 of 1)	Neg (0 of 2)	Yes	nd	b,li,lu,o,s	b,li,lu,o,s
	21	67	89	6	Neg (0 of 3)	Neg (0 of 2)	Neg (0 of 3)	Yes	nd	b,li,lu,o,s	b,li,lu,o,s
	22	68	79	8	Neg (0 of 4)	Neg (0 of 2)	Neg (0 of 4)	Yes	nd	b,li,lu,o,s	b,li,lu,o,s
	23	52	91	9	Neg (0 of 6)	Neg (0 of 2)	Neg (0 of 5)	Yes	s	b,li,lu,o,s	b,li,lu,o,s
	24	44	88	10	Neg (0 of 4)	Neg (0 of 2)	Neg (0 of 4)	Yes	nd	b,li,lu,s	b,li,lu,s
	25	42	76	12	Neg (0 of 5)	Neg (0 of 2)	Neg (0 of 5)	Yes	L.int,o,pln,S.int.,s	b,li,lu,o,s	b,li,lu,o,s
	26	63	88	12	Neg (0 of 7)	Neg (0 of 2)	Neg (0 of 6)	Yes	s	b,li,lu,o,s	b,li,lu,o,s
	27	42	88	12	Neg (0 of 7)	Neg (0 of 2)	Neg (0 of 6)	Yes	s	b,li,lu,o,s	b,li,lu,o,s
	28	38	86	15	Neg (0 of 8)	Neg (0 of 2)	Neg (0 of 8)	Yes	s	b,li,lu,o,s	b,li,lu,o,s
Mean (+/−SD)		51% (+/−17)	82% (+/−8)	9 (+/−4)							

* The data shown in the table includes analyses performed on both infected and uninfected mice with the text in bold used to highlight that HIV-1 was found in the indicated tissues. Numbers in parenthesis: first number represents the number of positive results out of the second number, which represents the number of different time points (total samples) tested. b – bone marrow; frt – female reproductive tract; li – liver; L.int – large intestine; lu – lung, mln – mesenteric lymph node; mrt – male reproductive tract; nd - not done; neg – negative; o – thymic organoid; pb – peripheral blood; pln – peripheral lymph node; pos – positive; r – rectum; S.int – small intestine; s – spleen; st – stomach.

A ELISA limit of detection = 7.8 pg/ml.

B Amplicor limit of detection = 400 copies/ml.

C Real-time PCR limit of detection = 10 copies.

**Figure 3:**
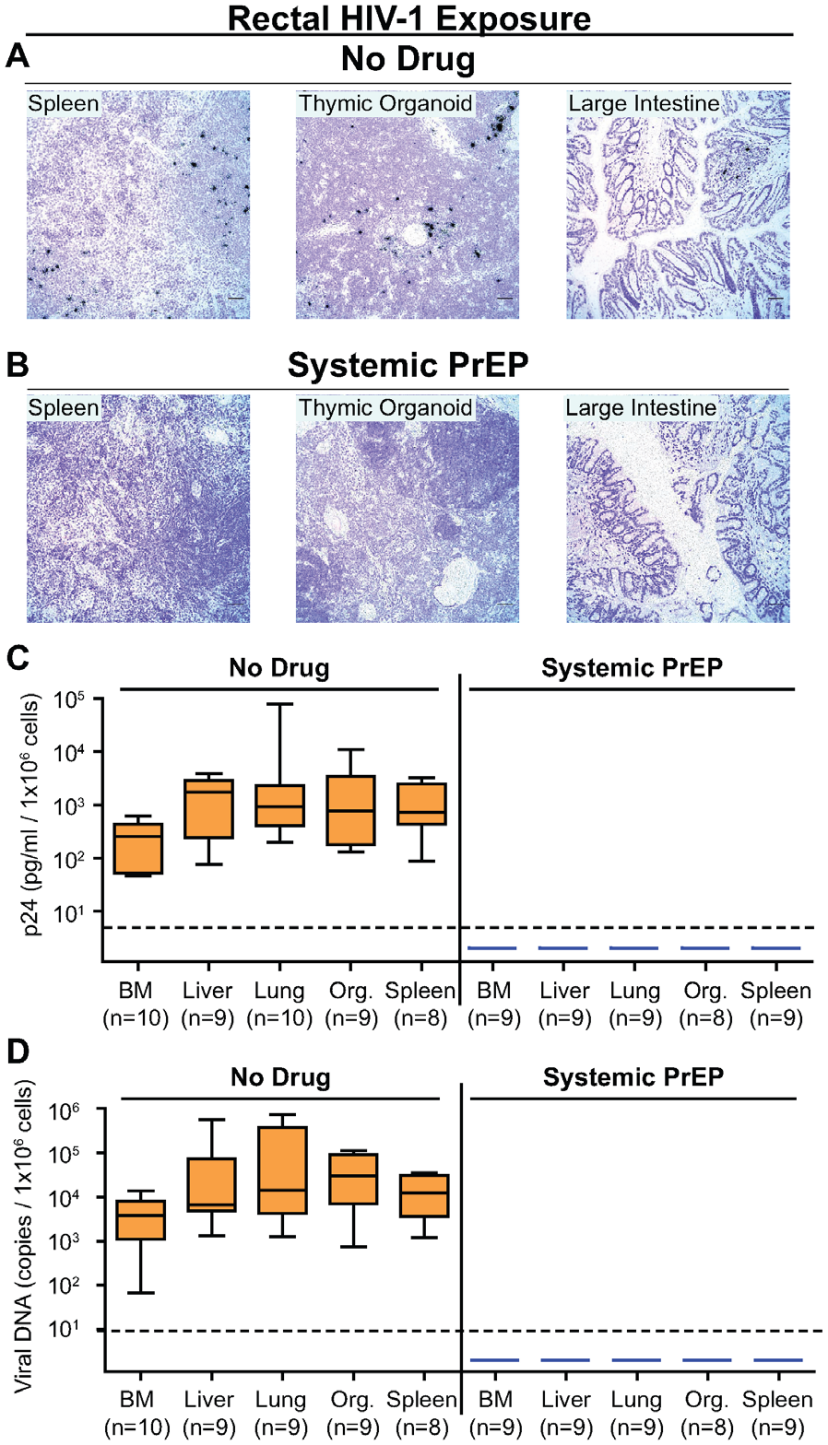
Systemic analyses of the protection afforded by FTC/TDF from rectal HIV-1 transmission. (A) Tissues from a representative non-treated control mouse (#1) showed the presence of productively HIV-1 infected cells expressing detectable viral RNA. (B) Tissues from a mouse receiving systemic PrEP (#25) demonstrated a complete lack of productively infected cells in any of the tissues analyzed. Black foci represent cells producing viral RNA (bar = 50 µm). (C) Tissues from infected non-treated control mice were positive for replication competent HIV-1 when co-cultured with activated allogeneic PBMC. Tissues from mice receiving systemic PrEP were consistently negative for the presence of HIV-1. Presence of replication competent virus was indicated by the detection of viral p24 in the culture supernatant. (D) Tissues from infected non-treated control mice were positive for HIV-1 DNA by real time PCR analysis. Tissues from mice that received systemic PrEP were consistently negative for the presence of HIV-1 DNA. Thin dashed lines represent the limit of detection for the respective assays.

We confirmed the lack of rectal HIV-1 transmission in BLT mice treated systemically with FTC/TDF using a comprehensive set of highly sensitive analytical techniques aimed at detecting the presence of HIV-1 in tissues. Specifically, we analyzed several tissues from these mice for evidence of viral RNA expression (in situ hybridization), replication competent virus (co-culture with activated allogeneic PBMC) or viral DNA (real time PCR). All tissues analyzed by each method for each mouse are detailed in Table 1. Whereas HIV-1 infection was confirmed by in situ hybridization for the presence of productively infected cells in tissues from the infected non-treated control mice, no productively infected cells were detected in the tissues obtained from the systemic PrEP treated mice (Figure 3A & B; Table 1). We also tested for the presence of cells containing replication competent HIV-1 with a co-culture virus rescue assay utilizing PHA/IL2 activated allogeneic PBMC. Whereas virus was rescued from cells originating in the tissues of infected non-treated mice, no virus was rescued from any tissues from the protected mice treated with antiretrovirals (Figure 3C; Table 1). Finally, real time PCR analysis of DNA obtained from cells isolated from tissues of infected non-treated control mice demonstrated the presence of viral DNA. In contrast, none of the mice receiving systemically applied antiretroviral PrEP exhibited viral DNA in tissues (Figure 3D; Table 1). In summary, these results demonstrate the absence of any evidence of HIV infection following systemic administration of antiretrovirals prior to exposure in humanized BLT mice.

### Systemic Administration of Antiretrovirals Results in Protection from Intravenous HIV-1 Infection

Having established the ability of systemic PrEP to prevent mucosal HIV-1 transmission, we sought to determine whether systemic PrEP could also prevent intravenous HIV-1 infection. During intravenous exposure no mucosal surfaces must be overcome by the virus in order to establish infection. Therefore, infection can potentially be established simultaneously in numerous sites throughout the body rendering protection from intravenous HIV-1 exposure much more difficult to achieve. We assessed the efficacy of systemically applied antiretrovirals to prevent intravenous HIV-1 transmission in BLT mice by administering a seven-day course of systemic PrEP with FTC/TDF as described previously for the vaginal and above for the rectal exposure experiments (Figure 1A) [24]. We exposed BLT mice (n = 8) intravenously to a single dose of HIV-1_JR-CSF_ 3 hours after the administration of the third of 7 consecutive daily doses of FTC/TDF (Figure 1A).

**Figure 4:**
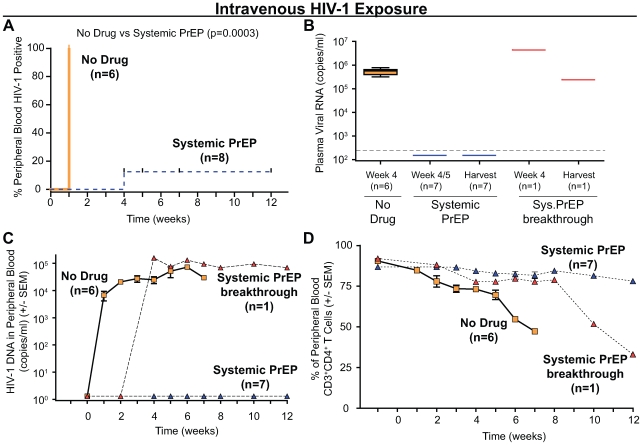
Systemic PrEP with FTC/TDF results in effective protection from intravenous HIV-1 transmission. (A) Kaplan-Meier plot of the time course to peripheral blood conversion following intravenous exposure to HIV-1 in BLT mice with or without systemic PrEP. (B) Seven (out of eight) mice receiving systemic PrEP were consistently negative for plasma viral RNA. Plasma viral RNA was detected in the systemic PrEP breakthrough mouse (#42) and the 6 non-treated control mice. Thin dashed line represents the limit of detection for this assay. (C) BLT mice receiving systemic PrEP were negative for PBMC-associated viral DNA by real time PCR. PBMC-associated viral DNA was detected in the systemic PrEP breakthrough mouse (#42) and the 6 non-treated control mice. (D) Average levels of human CD4^+^ T cells in peripheral blood showed loss of CD4^+^ T cells in the systemic PrEP breakthrough mouse (#42) and the 6 non-treated control mice. CD4^+^ T cells remained constant in the protected systemic PrEP treated BLT mice.

**Figure 5:**
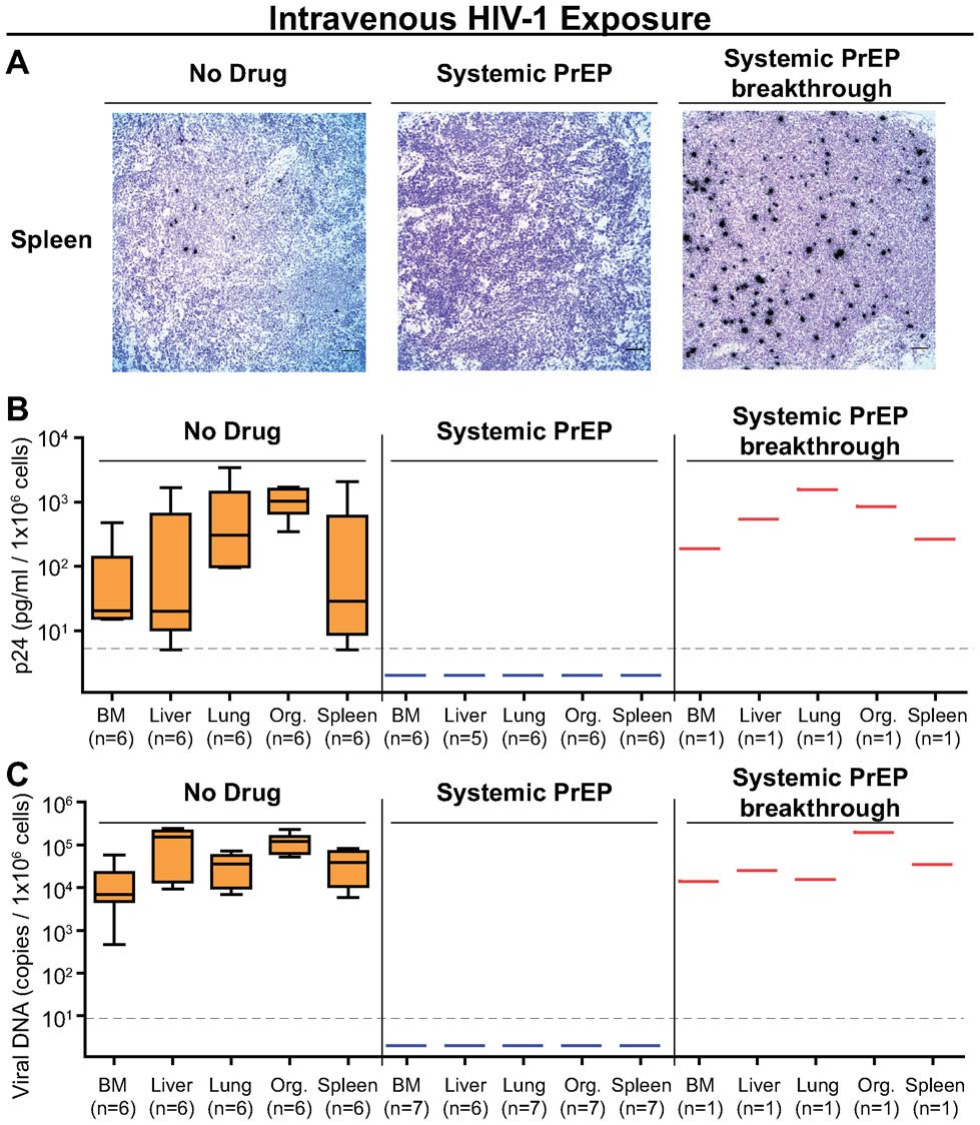
Systemic analyses of the protection afforded by FTC/TDF from intravenous HIV-1 transmission. (A) In situ hybridization analysis in spleens showed productively infected cells in a representative non-treated control mouse (#29) and the systemic PrEP breakthrough mouse (#42). In contrast, no productively infected cells were identified in the spleen of a representative systemic PrEP protected mouse (#38). Black foci represent cells producing viral RNA (bar = 50 µm). (B) Tissues from mice protected by systemic PrEP were consistently negative for the presence of HIV-1 when co-cultured with activated allogeneic PBMC. Replication competent HIV-1 was detected in the systemic PrEP breakthrough mouse (#42) and the 6 non-treated control mice. Presence of replication competent virus is indicated by the detection of viral p24 in the culture supernatant. (C) Tissues from mice given systemic PrEP were consistently negative for the presence of HIV-1 DNA by real time PCR. HIV-1 DNA was detected in the systemic PrEP breakthrough mouse (#42) and the 6 non-treated control mice. Thin dashed lines represent the limit of detection for the respective assays.

Consistent with the high efficiency of transmission associated with this type of exposure, in the absence of treatment we observed 100% transmission after intravenous inoculation (6/6) (Figure 4A–D; Table 2). In addition, we observed that intravenous infection could be delayed, but not prevented, when BLT mice (n = 4) were administered the 7 day treatment with FTC/TDF 24 hours following intravenous exposure to the same dose of HIV-1 (Log-rank [Mantel Cox] Test: Chi square = 9, df = 1, p = 0.003) (Table 2). Sequence analysis of the entire reverse transcriptase gene from these treated, infected mice demonstrated the absence of mutations associated with resistance to either FTC or TDF [37]. All tissues analyzed by each method for each of the mice used for these experiments are detailed in Table 2. In all cases, infection was confirmed by every criterion utilized (Figures 4 and 5; Table 2).

**Table 2:** Description of BLT mice used to evaluate systemic PrEP for intravenous HIV-1 transmission.*

Mouse #		In PB at exposure:		Weeks followed (exposure to harvest)	Peripheral Blood Analysis				Multiple Tissue Analysis		
		% human CD45^+^	% hCD45^+^ hCD3^+^ hCD4^+^		Plasma antigenemia (Gag^p24^)^A^	Plasma viral load (RNA)^B^	PBMC associated viral DNA^C^	Included in Figure 4B–D	In situ hybridization for viral RNA	Quantitative Real time PCR for cell associated viral DNA^C^	Virus rescue of replication competent virus (Gag^p24^)^A^
**No Drug**	29	77	91	4	**Pos (4 of 4)**	**Pos (1 of 1)**	**Pos (4 of 4)**	Yes	**c,L.int,lu,mrt,o,pln,s,ti**	**b,li,lu,o,s**	**b,li,lu,o,s**
	30	70	91	4	**Pos (4 of 4)**	**Pos (1 of 1)**	**Pos (4 of 4)**	Yes	**s**	**b,li,lu,o,s**	**b,li,lu,o,s**
	31	45	94	4	**Pos (4 of 4)**	**Pos (1 of 1)**	**Pos (4 of 4)**	Yes	**s**	**b,li,lu,o,s**	**b,li,lu,o,s**
	32	24	86	4	**Pos (2 of 4)**	**Pos (1 of 1)**	**Pos (4 of 4)**	Yes	**s**	**b,li,lu,o,s**	**b,li,lu,o,s**
	33	40	95	5	**Pos (5 of 5)**	**Pos (1 of 1)**	**Pos (4 of 4)**	Yes	nd	**b,li,lu,o,s**	**b,li,lu,o,s**
	34	31	86	7	**Pos (4 of 7)**	**Pos (1 of 1)**	**Pos (7 of 7)**	Yes	**s**	**b,li,lu,o,s**	**b,li,lu,o,s**
**FTC/TDF**	35	68	92	5	Neg (0 of 3)	Neg (0 of 2)	Neg (0 of 2)	Yes	s	b,li,lu,o,s	nd
	36	40	87	7	Neg (0 of 6)	Neg (0 of 2)	Neg (0 of 4)	Yes	nd	b,li,lu,o,s	b,li,lu,o,s
	37	50	84	7	Neg (0 of 6)	Neg (0 of 2)	Neg (0 of 4)	Yes	lu,o,pln,s	b,li,lu,o,s	b,li,lu,o,s
	38	58	83	7	Neg (0 of 6)	Neg (0 of 2)	Neg (0 of 4)	Yes	L.int.,lu,o,pln,S.int,s	b,li,lu,o,s	b,li,lu,o,s
	39	58	85	7	Neg (0 of 6)	Neg (0 of 2)	Neg (0 of 4)	Yes	lu,o,pln,s	b,li,lu,o,s	b,li,lu,o,s
	40	76	89	12	Neg (0 of 7)	Neg (0 of 2)	Neg (0 of 7)	Yes	mln,lu,pln,s	b,li,lu,o,s	b,li,lu,o,s
	41	75	88	12	Neg (0 of 7)	Neg (0 of 2)	Neg (0 of 7)	Yes	s	b,li,lu,o,s	b,li,lu,o,s
	42	67	92	12	**Pos (7 of 8)**	**Pos (2 of 2)**	**Pos (7 of 8)**	Yes	**s**	**b,li,lu,o,s**	**b,li,lu,o,s**
**FTC/TDF 24hr post**	43	56	96	3	**Pos (1 of 3)**	**Pos (1 of 1)**	**Pos (1 of 3)**	No	**s**	**b,li,lu,o,s**	**b,li,lu,o,s**
	44	30	83	4	**Pos (2 of 4)**	**Pos (1 of 1)**	**Pos (2 of 4)**	No	**s**	**b,li,lu,o,s**	**b,li,lu,o,s**
	45	36	94	7	**Pos (4 of 7)**	**Pos (1 of 1)**	**Pos (5 of 7)**	No	nd	**b,li,lu,o,s**	**b,li,lu,o,s**
	46	41	88	7	**Pos (5 of 7)**	**Pos (1 of 1)**	**Pos (5 of 7)**	No	**s**	**b,li,lu,o,s**	**b,li,lu,o,s**
Mean (+/−SD)		52% (+/−17)	89% (+/−4)	7 (+/−3)							

* The data shown in the table includes analyses performed on both infected and uninfected mice with the text in bold used to highlight that HIV-1 was found in the indicated tissues. Numbers in parenthesis: first number represents the number of positive results out of the second number, which represents the number of different time points (total samples) tested. b – bone marrow; c—caecum; li – liver; L.int – large intestine; lu – lung, mln – mesenteric lymph node; mrt – male reproductive tract; nd - not done; neg – negative; o – thymic organoid; pb – peripheral blood; pln – peripheral lymph node; pos – positive; S.int – small intestine; s – spleen; ti—terminal ileum.

A ELISA limit of detection = 7.8 pg/ml.

B Amplicor limit of detection = 400 copies/ml.

C Real-time PCR limit of detection = 10 copies.

In sharp contrast with the results described above, we observed protection from intravenous infection in 7 of 8 BLT mice that received the seven day course of systemic PrEP with FTC/TDF. Protection was determined by each of the following criteria: the lack of HIV-1 plasma antigenemia, PBMC-associated viral DNA, loss of CD4^+^ T cells or plasma viral RNA (Log-rank [Mantel Cox] Test: Chi square = 13, df = 1, p = 0.0003) (Figure 4A–D; Table 2). Comprehensive analyses of different tissues from these mice further confirmed that they were indeed fully protected from infection (Figure 5A–C, all tissues analyzed by each method are detailed in Table 2). In the case of the single breakthrough transmission (mouse #42), sequence analysis of the entire reverse transcriptase gene indicated that transmission was not due to the development of drug resistance [37]. Together, these results demonstrated that systemic administration of FTC/TDF PrEP can efficiently prevent intravenous infection with HIV-1 and illustrates the significant potential of PrEP to prevent intravenous HIV transmission in humans.

## Discussion

In this manuscript, we provide *in vivo* preclinical evidence supporting the hypothesis that systemic antiretroviral PrEP can provide broad protection from HIV transmission. Our results obtained using a highly relevant *in vivo* model of HIV transmission show that systemic antiretroviral PrEP can effectively prevent rectal and intravenous HIV-1 infection. It is important to note that systemic antiretroviral PrEP with a single drug combination prevents infection of BLT mice by the three most common routes of human HIV-1 transmission. The highly encouraging results from this comprehensive evaluation of antiretroviral PrEP efficacy serve as strong proof of principle for this approach and have major implications for the continued planning and implementation of future and current PrEP studies.

Approaches aimed at obtaining protection from all potential modes of transmission are highly significant. Individually, unprotected vaginal intercourse accounts for the vast majority of new HIV transmissions globally [40]. Rectal HIV exposure accounts for the majority of HIV transmission events in the United States and other developed nations [41]. Even though rectal HIV exposure is the main mode of transmission among men who have sex with men, this route is also likely to account for a significant number of transmissions to women [27]. Intravenous HIV exposure occurs primarily among injection drug users and is a growing health concern in many nations [42]. Unlike mucosal exposure with its intrinsic physical and biological barriers, the direct exposure of virus to the blood stream results in more efficient transmission. Our results provide clear pre-clinical evidence of the potential usefulness of systemic PrEP for intravenous transmission. The ability to prevent HIV-1 transmission by all three routes using one drug combination has the potential of greatly facilitating the global implementation of preventative measures.

Until this study, *in vivo* preclinical data substantiating a broad prevention approach using a single drug combination to prevent three routes of transmission had been lacking. *In vivo* data on the efficacy of PrEP with FTC/TDF had been limited to two reports relating to mucosal transmission. In one study, we showed that systemic PrEP with FTC/TDF can effectively prevent vaginal HIV-1 transmission in BLT mice [24]. The second study used rhesus macaques to show that intermittent or daily systemic PrEP with FTC/TDF can protect from rectal SHIV transmission in a low-dose repeat exposure model [43]. Collectively, these two reports and the current data show that antiretroviral PrEP with FTC/TDF can afford extensive protection from vaginal, rectal and intravenous HIV-1 transmission.

When considering such broad use of antiretrovirals as prophylaxis, there is an issue of major importance that must be addressed. In humans, lack of strict compliance to PrEP regimens could increase the likelihood of drug resistance being developed in the event of breakthrough infection. Therefore broad antiretroviral use can result in increased emergence of resistance to the drug(s) when infections do occur [11]. Spread of resistant viruses could limit the efficacy of current therapeutic interventions using these same drugs, although it should be noted that the fitness of multidrug resistant viruses for mucosal transmission has yet to be fully established. Future BLT mouse studies could model lack of compliance to evaluate the fitness of multidrug resistant viruses for mucosal transmission and explore potential mechanisms of breakthrough infections. Despite the high protection observed while using PrEP (>88%), our results indicated one breakthrough infection observed in one animal infected intravenously. It should be noted that sequence analysis of the entire reverse transcriptase gene revealed that this one transmission event was not the direct result of the appearance of mutations associated with drug-resistance [37]. The molecular basis for transmission of wild type virus after venous exposure in the presence of PrEP remains to be determined.

Results obtained using humanized BLT mice must be considered in the context of previous studies of antiretrovirals for HIV prevention performed in other models such as non-human primates. Experiments performed using non-human primates have provided evidence for the use of tenofovir (PMPA) to prevent intravenous infection by SIV_mne_ in long-tailed macaques [44] and successful antiretroviral PrEP in rhesus macaques exposed rectally to either SIV_mac251/32H_ or SHIV_SF162P3_ have also been reported with this compound [45], [46]. Topical and systemic PrEP with one or more fusion inhibitors protected from vaginal SHIV transmission in rhesus macaques [47], [48]. Systemic PrEP with FTC/TDF was shown to prevent rectal SHIV transmission in rhesus macaques [43] and in yet another non-human primate model, 2 pig-tailed macaques were protected from intravenous challenge with simian-tropic HIV (stHIV) by systemic PrEP with efavirenz plus FTC/TDF [49]. Additional preclinical studies in macaques testing antiretroviral HIV-1 prevention modalities have focused on post-exposure prophylaxis, not pre-exposure regimens [44], [50]–[54]. The use of multiple animal models and different classes/combinations of drugs in these studies makes it difficult to make direct comparisons and to extrapolate potential outcomes. The current study represents a significant advance because it has produced a data set that can be easily interpreted and easily compared across multiple virus transmission routes all within the same experimental platform.

While our findings and those from non-human primate research suggest that antiretroviral PrEP can prevent HIV transmission, neither model has been shown to predict efficacy or safety in humans. This limitation exists because there is still no evidence of efficacy for antiretrovirals in preventing vaginal, rectal or intravenous transmission in humans [7], [10], [12], [15], [55]. It will be essential that ongoing human clinical trial data be compared to BLT and non-human primate studies in order to validate these useful models. Protection is likely to be dependent on the drug exposure levels achieved following dosing of the PrEP antiretrovirals. Currently, there is no comparative pharmacological data of these levels between humans and BLT mice. Since protection is dependent on the dose of FTC/TDF it will be important to define the drug exposure of the regimen in BLT mice, and assess its relationship to the drug exposure achieved after oral dosing with Truvada in humans. Detailed information on the drug exposure in BLT mice will be important for interpreting the efficacy results in this model and for comparison with efficacy data from human trials when these become available in the near future. These data might help assess the relationship between drug exposure achieved after oral dosing with Truvada in humans and its effectiveness in ongoing clinical trials. In addition, a significant strength of humanized BLT mice is the fact that they can be used in future studies to address other potential variables between BLT mice and humans including differences in timing of dosing, drug concentrations, adherence to drug regimens, virus inoculum and relevant co-infections. Our results also suggest that humanized BLT mice will be useful for the evaluation of topical microbicides and to provide preclinical evidence for their potential success. The availability of a small animal model such as BLT mice for screening prevention modalities prior to or in conjunction with macaque and human studies is a great asset to the field [56].

In conclusion, we provide preclinical evidence regarding the potential efficacy of an antiretroviral pre-exposure prophylactic approach to prevent vaginal, rectal and intravenous HIV-1 transmission. Our results provide strong support for the continued implementation of clinical trials using targeted antiretroviral pre-exposure prophylaxis for all the major routes of HIV transmission contributing to the HIV/AIDS pandemic.
